# Supplementary material for: A Novel Earwax Method to Measure Acute and Chronic Glucose Levels
Source: Diagnostics (Basel). 2020 Dec 10;10(12):1069. doi: 10.3390/diagnostics10121069 (PMC7764152; doi:10.3390/diagnostics10121069)
Supplement: Supplementary file 1 [file diagnostics-10-01069-s001.pdf]

Supplementary Methods

Earwax Sample Extraction

A clinical research assistant was specifically trained in the use of the Reiner-Alexander syringe by one ear-nose-throat specialist doctor. Before cleaning both ears, the external auditory canal was examined using an otoscope to rule out the presence of any external ear pathology, such as impacted earwax or perforated eardrum. Briefly, the Reiner-Alexander syringe slowly injects water at 37°C inside the external ear canal. The process of syringing creates a sensation of mild pressure in the ear as the warm water from the syringe flushes the wax out. The expelled water and the extracted earwax secretion were collected in a kidney basin. During the follow-up visit, participants self-clean their right ears using the earwax self-sampling device, according to the manufacturer instructions (www.trears.com). The four labelled earwax samples were stored at 4 °C until they were analysed.

Earwax Analysis using the Reiner-Alexander Syringe

The extracted solution of water plus earwax secretion was stored in a 50 ml cryovial. Earwax samples were then resuspended with 500 µl Phosphate-Buffered Saline (PBS) to homogenise the sample. Then, 500 µl of diethyl ether was added to each sample and wobbled for one minute using a vortex. The resulting solution was stored at -20°C for 2 hours. Glucose levels were then analysed from the hydrophilic fraction. The earwax solution was dried using the displacement method of N_2 at 25 degree Celsius. Following this, 125 µl of PPBS was added to resuspend each solution. The resulting solution containing earwax samples was stored at 4 degrees Celsius until analysed.

Earwax Analysis using the earwax self-sampling device

The earwax self-sampling device tips were cleaned using 500 µl of PBS for 2 minutes. Then, the same previous steps that were done for analysing earwax sample using the metallic syringe were followed when the novel device was also used.

Blood Samples:

All blood samples, baseline and follow up, for every participant, were taken in the morning. Blood samples were taken from the cephalic vein using a 3cc syringe without using any anticoagulants. Participants had been previously instructed to avoid eating or drinking anything 8 hours before the baseline assessment. Another blood sample was taken during the morning of the follow-up visit. The follow-up samples were taken 2 hours after consuming a standardised liquid meal, 236 ml of Ensure Avance®. This meal contains 1.5 kcal/ml, given by 24.3% protein, 44.8% carbohydrate 28.8% fat, 1% fibre and 1.1% Beta-hydroxy-beta-methyl butyrate. FSG and HbA_{1c} levels were analysed from baseline samples. HbA_{1c} levels and PSG were analysed from the follow-up samples. The chronic glucose level over the preceding one-month period was calculated using the mean between the baseline and the follow-up sample of glycaemia.

Serum Glucose Analysis:

Blood samples were coagulated after being stored at 4°C for 24-h. It allows the separation of the serum fraction from the clotting factors, such as fibrinogen. Following the separation, serum samples

were centrifugated (1000 x g) for 20 min at 4°C. The resulting pellet was separated from the solution. The solution was recollected in labelled tubes of 2 ml. These tubes were stored -20°C until their content was analysed.

Glucose Analysis using Serum and Earwax Samples

Glucose levels were measured using enzymatic oxidation testing and the labelling of oxidized glucose using 96-well plates, according to manufacturer instructions (BioVision Inc., Milpitas, CA, USA). Diluted standard assay with 1:25 of serum were added to the wells including 50 µl of a mixed portion of glucose which contains 2 µl of probed glucose and 2 µl of an enzymatic mix for glucose. The resulting solution was incubated for 30 min at 37°C and protected from direct light. Absorbance was read at 570 nm using a microplate reader (NovoStar).

Haemoglobin analysis

Total haemoglobin was measured using the standard colorimetric method of cyanmethemoglobin, according to the manufacturer instructions (Valtek Laboratory, Santiago, Chile). Briefly, 1 µl of blood were incubated for 10 minutes with 250 µl of Drabkin solution at room temperature. Sample absorbances were measured at 540 nm using a microplate reader (NovoStar).

Glycated Haemoglobin Analysis:

HbA_{1c} was measured using a sandwich ELISA technique, according to the manufacturer's instructions (Abbexa Ltd., Cambridge, UK). 100 µl of the diluted standards were added into the standard wells. An antibody, specific to $HbA_{1c,}$ is already precoated onto the 96 well plates. A competitive inhibition reaction was initiated between biotin labelled HbA_{1c} and unlabelled HbA_{1c} with the pre-coated antibody specific to HbA_{1c}. The unbound conjugates were washed away 3 times. Following this, avidin conjugated to Horseradish Peroxidase was added to each microplate well and incubated. 3,3′,5,5′-Tetramethylbenzidine (TMB) Substrate Buffer substrate solution was then added. The wells that contained HbA1c, underwent a colour change, from a blue coloured product yellow, after adding acidic stop solution. The intensity of the yellow colour was inversely proportional to the HbA_{1c} amount bound on the plate. The optical density absorbance was measured spectrophotometrically at 450 nm in a microplate reader, and the concentration of HbA_{1c} was calculated. % HbA_{1c} was calculated using the National Glycohemoglobin Standardization Program (NGSP) units which express the percentage of HbA_{1c} over the total amount of haemoglobin.
